# Is There a G Factor for Metacognition? Correlations in Retrospective Metacognitive Sensitivity Across Tasks

**DOI:** 10.1037/xge0000746

**Published:** 2020-03-19

**Authors:** Audrey Mazancieux, Stephen M. Fleming, Céline Souchay, Chris J. A. Moulin

**Affiliations:** 1 Laboratory of Psychology and Neurocognition, Scientific Research National Center 5105, Grenoble Alpes University; 2 Wellcome Centre for Human Neuroimaging and Max Planck UCL Centre for Computational Psychiatry and Ageing, University College London; 3 Laboratory of Psychology and Neurocognition, Scientific Research National Center 5105, Grenoble Alpes University

**Keywords:** metacognition, metamemory, monitoring, confidence judgments, domain-general processes

## Abstract

Is metacognition a general resource shared across domains? Previous research has documented consistent biases in judgments across tasks. In contrast, there is debate regarding the domain generality or the domain specificity of the ability to discriminate between correct and incorrect answers (metacognitive sensitivity) because most previous work has documented nonsignificant correlations across domains. However, such null findings may be due to low statistical power and differences in task structure or performance, thereby masking a latent domain generality in metacognition. We examined across-domain correlations in confidence level and sensitivity in a large sample (*N* = 181). Participants performed 4 2-alternative forced-choice tasks (episodic memory, semantic memory, executive function, and visual perception) with trial-by-trial confidence judgments. We found significant correlations in average confidence level across tasks. By applying a hierarchical Bayesian model to estimate cross-task covariance, we found five out 6 cross-task correlations in metacognitive efficiency (meta-*d*′/*d*′) were significant, even for pairs of tasks in which first-order performance was not correlated. This suggests that at least some components of metacognitive efficiency in retrospective confidence are domain general.

Metacognition refers to the ability to monitor and control cognitive processes (Flavell, 1979). It is often studied with reference to memory (e.g., Nelson & Narens, 1990) but has also recently been quantified for other domains such as visual perception (e.g., Song et al., 2011), decision making (e.g., Yeung & Summerfield, 2012), and motor tasks (e.g., Simon & Bjork, 2001). A critical research question therefore concerns the cross-domain organization of such metacognitive evaluations of cognition. The core question of this article is whether metacognition is a specific process particular to each cognitive domain (e.g., language, memory, perception) or whether it is a higher-order process with some overlap across multiple cognitive domains. A domain-general view of metacognition proposes that people use a common resource when they evaluate their performance across different types of tasks. In contrast, a domain-specific account proposes that there are different metacognitive components at play in different tasks.

By leveraging individual differences, it is possible to adjudicate between these two proposals. According to the domain-general view, people who have accurate judgments for one task should also make accurate judgments for another. In contrast, if metacognition relies on domain-specific components, we would expect such abilities to be uncorrelated. The focus of this paper is to investigate this issue using retrospective confidence judgments (RCJs). RCJs are self-evaluations of certainty in a given response and are appropriate for addressing the question of domain generality because they can be applied to decisions made across a variety of tasks.

In the current study, we focus on assessing the domain generality of both metacognitive bias and sensitivity, two measures that map onto two different aspects of metacognition. Metacognitive bias refers to the overall magnitude of a judgment, such as whether an observer has a tendency to report high or low confidence, irrespective of their performance. Metacognitive sensitivity refers to the ability of a person to discriminate between different levels of performance, such as correct or incorrect trials (Fleming & Lau, 2014).

Previous research using RCJs has provided equivocal findings for metacognitive sensitivity. Whereas a few studies have found positive correlations between metacognitive sensitivity for memory and visual perception tasks (McCurdy et al., 2013; Lee, Ruby, Giles, & Lau, 2018), a majority concluded in favor of domain specificity because of nonsignificant correlations (Baird, Cieslak, Smallwood, Grafton, & Schooler, 2015; Baird, Smallwood, Gorgolewski, & Margulies, 2013; Fitzgerald, Arvaneh, & Dockree, 2017; Morales, Lau, & Fleming, 2018). Regarding structural magnetic resonance imaging data, distinct cerebral areas correlating with individual variation within two tasks has been observed, also supporting the possibility of neurofunctional independence between domains (Baird et al., 2013; Baird et al., 2015; McCurdy et al., 2013). Specifically, metacognitive sensitivity in a visual perception task has been related to the volume and function of lateral anterior prefrontal cortex (aPFC), whereas metacognitive sensitivity in a memory task is associated with the structure and function of precuneus and medial aPFC. Accordingly, lesions to aPFC have been shown to selectively affect visual perceptual sensitivity while sparing sensitivity on the memory task (Fleming, Ryu, Golfinos, & Blackmon, 2014).

However, a recent meta-analysis of cross-domain correlations in metacognitive sensitivity pointed to a heterogeneous pattern of domain generality (Rouault, McWilliams, Allen, & Fleming, 2018). Although there was an overall cross-domain correlation between different perceptual tasks (e.g., visual, auditory, tactile; see, for instance, Ais, Zylberberg, Barttfeld, & Sigman, 2016; Faivre, Filevich, Solovey, Kühn, & Blanke, 2018), there was equivocal evidence for domain generality across visual perception and memory tasks. Moreover, it was noted that drawing conclusions about domain-specificity relies on accepting the null hypothesis of no correlation, which is problematic if individual experiments are underpowered to detect a correlation. In addition, it was recognized that cross-domain correlations may also be biased by inconsistencies in the sensitivity index calculated in these studies and variability in task structure between domains.

A first important consideration is the method used to assess metacognitive sensitivity. Different techniques are often used to compute sensitivity, which makes it difficult to compare results across studies. Moreover, several of these indexes (such as gamma correlation or area under the type II receiver-operating characteristics (ROC); area under the Type II ROC curve [AUROC2]) do not control for the effect of task performance (Fleming et al., 2014), and spurious correlations in metacognitive sensitivity may emerge between domains that are driven by variation in task performance (i.e., first-order performance) rather than metacognitive capacity itself (i.e., second-order performance; Rouault et al., 2018). One recent measure that achieves this control is metacognitive efficiency, meta-*d*′/*d*′. The meta-*d*′ framework models the relationship between performance and metacognition using signal detection theory (SDT). Meta-*d*′ is defined as the type I *d*′ that would lead to the observed type II ROC curve in the absence of noise or imprecision in confidence estimates (Maniscalco & Lau, 2012). Metacognitive efficiency is then defined as the level of metacognitive sensitivity (meta-*d*′) of a subject relative to the subject’s actual type I performance. By estimating meta-*d*′ in a Bayesian hierarchical framework (Fleming, 2017), it is possible to directly estimate covariance in metacognitive efficiencies across domains.

A second possible explanation for inconsistencies between results of previous studies is that different task designs have been used in different domains. For instance, several studies have compared metacognitive sensitivity between 2 alternative forced choice (2AFC) perceptual tasks and yes/no recognition memory tasks. As recently suggested (Lee et al., 2018), these differences in task structure may obscure across-domain correlations in metacognitive ability, particularly given potential asymmetries in metacognitive ability for yes and no responses (Kanai, Walsh, & Tseng, 2010; Meuwese, van Loon, Lamme, & Fahrenfort, 2014). Here we focus on comparing between different 2AFC tasks that are appropriate for fitting an equal-variance meta-*d*′ model.

Unlike the debate surrounding metacognitive sensitivity, there is greater agreement in previous literature that metacognitive bias is relatively stable across tasks. People tend to be overconfident in their judgments of general knowledge (Lichtenstein & Fischhoff, 1977) and visual perception (Baranski & Petrusic, 1994; Song et al., 2011), and this degree of confidence is correlated across tasks (Ais et al., 2016). Moreover, the hard-easy effect—overestimation in difficult tasks and underestimation in easy tasks—has also been found in both types of task (e.g., Baranski & Petrusic, 1995). In sum, whereas previous studies support a domain generality in metacognitive bias, both neuroimaging and behavioral findings, albeit in small samples, remain equivocal about the domain generality of metacognitive sensitivity.

On a theoretical level, models of metacognition have been developed in two distinct fields: metamemory (metacognition about memory) and metaperception (metacognition about perceptual decision making). Although these frameworks have developed independently, common points can be highlighted. Models of confidence formation in perceptual decision making suggest that confidence is based on a computation of a probability that a decision is correct. A dominant view supports the idea that confidence relies on both evidence from the first-order decision and additional computations beyond this such as postdecisional processes (Navajas, Bahrami, & Latham, 2016) or second-order inference (Fleming & Daw, 2017).

Similarly, in metamemory, the amount and quality of evidence is proposed to be critical in supporting a confidence estimate (e.g., Koriat, Lichtenstein, & Fischhoff, 1980). One component of such evidence are cues that are intrinsically related to memory processes (e.g., extrinsic information such as number of stimuli to encode, relatedness between targets and distractors, Koriat, 1997), equivalent to the notion of sensory evidence in perceptual decision making. However, as in metaperception, metamemory confidence (and other metacognitive judgments) is thought to also be inferred from additional information that may not be used to guide first-order memory responses. In the metaperception field, confidence has been modeled using extensions of SDT and evidence accumulation frameworks, whereas the computational distinction between first- and second-order processes in memory has received less attention. For instance, according to the stochastic detection and retrieval model (Jang, Wallsten, & Huber, 2012), a first sample of evidence informs a recall or recognition response and a second sample of evidence supports the formation of confidence. This model, as in related models of perceptual confidence (Fleming et al., 2017), suggests that additional computations (that can more or less correlated with a first-order decision computation) are used to inform confidence judgments. It is therefore possible that both domain-specific (i.e., internal perceptual or mnemonic states supporting first-order decisions in each task) and domain-general resources (i.e., postdecisional computations that could be common across tasks) contribute to confidence judgments in the two domains.

Motivated by these theoretical issues, the aim of the present study was to compare metacognitive judgments across four different 2AFC cognitive tasks and to ask whether correlations in bias (measured by confidence level) and/or sensitivity (measured by meta-*d*′) are indicative of a common underlying process of metacognition. The idea was to quantify potential domain-general contributions to metacognition while keeping the task structure similar across first-order decisions. As noted above, it remains possible that an absence of correlations regarding metacognitive sensitivity is explained by a lack of statistical power because the sample sizes of previously mentioned studies ranged from 23 to 52 participants. It is, however, important to note that these studies are mainly neuroimaging studies that did not directly aim to test cross-task correlations in behavioral measures of metacognition. To test a correlation hypothesis, it has been suggested that “there are few occasions in which it may be justifiable to go below *n* = 150” to obtain stable and reliable correlations (Schönbrodt & Perugini, 2013, p. 10). Here we use a large sample (*N* = 181) based on a priori power calculations and compute the covariance of meta-*d*′/*d*′ estimates in a hierarchical Bayesian framework, thereby maximizing the sensitivity of our analysis approach to detect shared variance across domains.

## Method

### Participants

The current experiment was conducted in the Laboratoire de Psychologie et Neurocognition in Grenoble, France, and included 181 young adults (*M* = 20.01, *SD* = 3.13; 84% of women) recruited through an advertisement at the Grenoble-Alpes University. We estimated the required sample size according to Schönbrodt et al. (2013) using an expected correlation of 0.4 between metacognitive sensitivity on a memory and a perceptual task (McCurdy et al., 2013). The authors explained that “the true correlation strength uncontaminated by outlier influence, although significant, is likely to be lower than the *r* value of 0.471” (p.4), hence our more conservative estimate of 0.4. According to Schönbrodt et al. (2013), for a correlation of 0.4 and 80% of power, correlations begin to be stable for 181 participants. All participants were native French speakers and reported having normal or corrected-to-normal vision. The study was preregistered on the Open Science Framework (https://osf.io/b5ype/) and preregistered analyses are presented in online supplemental material. We report here nonpreregistered analyses (see data and statistical analyses section).

### Materials and Procedure

**Figure 1 fig1:**
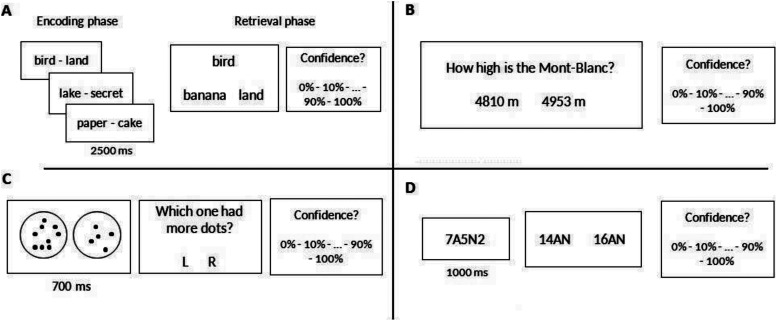
Summary of the four tasks. A, Episodic memory task. B, Semantic memory task. C, Visual perception task—real stimuli included between 25 and 75 dots. D, Working memory/attention task (executive functioning).

The entire procedure included four cognitive tasks: an episodic memory task, a semantic memory task, an executive functioning task, and a visual perception task. Task order was randomly assigned for each participant. See Figure 1 for examples and a schematic representation. The episodic memory task was separated into two parts: an encoding phase and a retrieval phase. During the encoding phase, participants were presented with 40 unrelated pairs of words for 2,500 ms duration in a randomized order. Words were extracted from the French Lexique database (New, Pallier, Brysbaert, & Ferrand, 2004) according to the following criteria: nouns or adjectives with six letters, two syllables, and between 20 and 100 occurrences per million. During the retrieval phase, immediately after the end of the encoding phase, participants were presented with a cue word seen during the encoding phase and had to select which one of the two other presented words was paired with this cue word. Participants had no time limit to give their answer. Distractors were other words extracted from Lexique according to the same criteria as targets and cues. These 2AFC decisions in this task, and in the following, are referred to as the firs-order task.[Fig-anchor fig1]

In the semantic memory task, participants performed a series of 2AFC decisions for general knowledge questions specifically designed for the French participants in this study. These questions included various topics such as cinema, sport, art, history, and geography (e.g., What is the largest department in France? Which painter is the main representative of Cubism?). We pretested the difficulty of 60 questions in 20 participants by calculating the percent correct for each question. From these 60 questions, 20 were excluded because they were either too easy (above 95% correct answers) or too difficult (bellow 5% correct answers). Participants had no time limit to give their answer.

The visual perception task was akin to the one used by Fleming et al. (2014) and consisted of two circles (diameter of 11.5°), each containing dots presented for 700 ms. After stimuli presentations, participants responded as to which one of the two circles contained more dots with no time limit. Before each new stimulus presentation, participants had to press the space bar. One of the two circles always contained 50 dots and the other either had fewer than or more than 50 dots, randomly defined on each trial. Stimuli were created using a plot function in R software. For each stimulus the number of dots was randomly defined—between 25 and 49 for stimuli with fewer dots and between 51 and 75 for stimuli with more dots.

The fourth task consisted of an attention, flexibility, and working memory (executive function) task. Participants were presented a letter-number sequence of five symbols for 1,000 ms. Half of these sequences had three letters and two numbers and the other half had two letters and three numbers (e.g., 7A5N2). Participants chose which one of the two presented responses corresponded to the sum of all numbers and the relevant letters (in the example above the correct answer would be 14AN). They had no time limit to give their answer and had to press the space bar before each new stimulus presentation. All stimuli were made prior to the task by associating random letters (from A to Z) with numbers (from 0 to 9). Distractors were made by changing either one letter or the sum of all numbers (e.g., if the correct response is 14AN, distractors can be either 16AN or 14BN) from the correct answer. All stimuli had the same structure with numbers embedded in strings of letters.

All four tasks comprised 40 trials each and had similar response requirements. The position of the correct answer was randomly assigned and the order of the four tasks was randomized for all participants. To begin each trial, participants pressed the space bar. For the first-order decision, participants had to press the s letter to select the left-hand answer and the l letter to select the righthand answer and they had no time limit for make their decision. Figure 1 provides a summary of the four tasks.

After each response on each of the four tasks, participants were asked to evaluate how confident they were in their answer. The scale ranged from 0% of confidence (minimum confidence) to 100% (maximum confidence). Participants could report 10%, 20%, 30%, 40%, 50%, 60%, 70%, 80%, and 90% by using the number keys 0 to 9. Participants used c to report 100% confidence. It was explained to the participants that 0% confidence signified a guess response. There was no time limit for either first-order decisions or confidence judgments and participants were not asked to respond as quickly as possible; however, we measured decision time in an exploratory analysis.

### Data and Statistical Analyses

As described above, we focused on both metacognitive bias and metacognitive sensitivity. In our initial preregistration, we aimed to measure metacognitive bias by subtracting mean task performance from mean confidence because we anticipated that first-order performance would differ across the four tasks. Metacognitive sensitivity was proposed to be measured by the area under the type II ROC curve. We decided to deviate from both of these planned analyses for several reasons (see online supplemental material for preregistered analyses).

Regarding metacognitive bias, we reasoned that there was some ambiguity in the absolute meaning of the scale label 0% confident, given that chance level in 2AFC tasks is 50%. We therefore decided to measure the average confidence level across trials without subtracting mean task performance, which would rely on subjects having interpreted a scale value of 0% confidence as 50% performance (chance).

We chose to estimate metacognitive efficiency (meta-*d*′/*d*′) – that is, metacognitive sensitivity corrected for differences in performance—when comparing cross-task correlations in metacognitive capacity. This is because measures of metacognitive sensitivity (such as gamma correlation and AUROC2) are sensitive to differences in first-order performance (e.g., Fleming et al., 2014), rendering such scores inappropriate for the current study in which task performance varied across both domains and participants. Using AUROC2, for instance, it is possible that cross-task correlations at the metacognitive level could be partly or fully driven by correlations in first-order performance. The meta-*d*′ framework allows us to control for such variability. In type I SDT, *d*′ refers to the ability to discriminate between different states of the world (i.e., signal and noise). This parameter can be calculated as *d*′ = z(hits) - z(false alarms), where z is the inverse of the cumulative normal distribution function, hits are the proportion of signal responses when signal is present, and false alarms are the proportion of signal responses when noise is present (here signal was defined arbitrarily as one of the two response options because two stimulus options were presented on each trial of the 2AFC tasks). In type II SDT, the sensitivity parameter of interest is the ability to discriminate between correct and incorrect responses, rather than signal and noise. Meta-*d*′ refers to the type I *d*′ that would give rise to the observed confidence distributions in the absence of noise or imprecision in the ratings. By modeling the relationship between type I and type II performance (the more information available for the type I task, the more sensitive type II confidence ratings should be), meta-*d*′ quantifies the sensitivity of confidence ratings to performance in units of *d*′ (Maniscalco et al., 2012). Because *d*′ and meta-*d*′ are in the same units, they can be compared, which allows derivation of a measure of metacognitive efficiency, controlling for task performance. If this measure (Mratio; meta-*d*′/*d*′) is close to 1, then metacognitive efficiency is optimal under the SDT model.

Here we used a recent hierarchical Bayesian framework (Fleming, 2017) to estimate meta-*d*′/*d*′ at the group level (HMeta-d). This allows a more accurate estimation of subject-level parameters by allowing the group-level estimates to constrain subject-level fits and more stable group-level estimates by limiting the impact of single-subject estimates with high uncertainty on the group. Fleming (2017) showed in simulation that HMeta-d was able to recover stable group-level parameter estimates with as few as 50 trials per subject, which was not the case when averaging single-subject maximum likelihood fits. This framework is also particularly useful to test the question of the domain generality of metacognition because it can also be used to estimate covariance between estimates in a hierarchical framework.

Because we have a low number of trial per task (*N* = 40), a Bayesian estimation of meta-*d*′ is more appropriate because it naturally handles zero cell counts and avoids the use of edge correction, which may bias maximum likelihood estimates. Moreover, maximum likelihood estimates of parameters based on hit and false alarm rates fail to take into account uncertainty about these rates that is a consequence of finite data. A Bayesian approach takes into account the uncertainty about single-subject parameter estimates at the group level and thus naturally handles both within- and between-participants uncertainty. This is particularly crucial in the current study, given that uncertainty in the model’s estimate of meta-*d*′ needs to be incorporated into an assessment of any correlation between the two domains (see online supplemental material).

To extend the existing model, each subject’s log metacognitive efficiency (log(meta-*d*′/*d*′)) in the four tasks (M1, M2, M3, M4) was specified as a draw from a multivariate Gaussian:

$$\left[\log\left(M1_{s}\right)\log\left(M2_{s}\right)\log\left(M3_{s}\right)\log\left(M4_{s}\right)\right]∼N\left(\left[\begin{array}{c} \begin{array}{c} \mu_{M1}\\ \mu_{M2}\\ \mu_{M3}\\ \mu_{M4} \end{array} \end{array}\right],\left[\begin{array}{c} \begin{array}{cccc} \sigma_{M1}^{2} & \rho_{M1M2}\sigma_{M1}\sigma_{M2} & \rho_{M1M3}\sigma_{M1}\sigma_{M3} & \rho_{M1M4}\sigma_{M1}\sigma_{M4}\\ \rho_{M1M2}\sigma_{M1}\sigma_{M2} & \sigma_{M2}^{2} & \rho_{M2M3}\sigma_{M2}\sigma_{M3} & \rho_{M2M4}\sigma_{M2}\sigma_{M4}\\ \rho_{M1M3}\sigma_{M1}\sigma_{M3} & \rho_{M2M3}\sigma_{M2}\sigma_{M3} & \sigma_{M3}^{2} & \rho_{M3M4}\sigma_{M3}\sigma_{M4}\\ \rho_{M1M4}\sigma_{M1}\sigma_{M4} & \rho_{M2M4}\sigma_{M2}\sigma_{M4} & \rho_{M3M4}\sigma_{M3}\sigma_{M4} & \sigma_{M4}^{2} \end{array} \end{array}\right]\right)$$

Priors were specified as follows:

$$\mu_{M1},\mu_{M2},\mu_{M3},\mu_{M4}∼N(0,1)$$

$$\sigma_{M1},\sigma_{M2},\sigma_{M3},\sigma_{M4}∼InvSqrtGamma(0.001,0.001)$$

$$\rho_{M1M2},\rho_{M1M3},\rho_{M1M4},\rho_{M2M3},\rho_{M2M4},\rho_{M3M4}∼Uniform(-1,1)$$

*N* is a normal distribution with mean and standard deviation as parameters. μ_*M*_ and σ_*M*_ refer to the mean and the standard deviation of log(meta-*d*′/*d*′). ρ_*MiMj*_ is the correlation coefficient for log(meta-*d*′/*d*′) between tasks *i* and *j*.

The HMeta-d toolbox (https://github.com/metacoglab/HMeta-d) uses Markov chain Monte Carlo sampling to estimate posterior distribution over model parameters using the JAGS program (Plummer, 2003). We modified the HMeta-d code to allow estimation of parameters in R using rjags. As in the HMeta-d toolbox, we discarded early samples of the posterior distributions and ran three chains to diagnose convergence problems. Convergence diagnostics were computed with the coda package using the potential scale reduction factor *R̂* (Gelman & Rubin, 1992). Material, raw data, model, and analysis scripts are available in OSF (https://osf.io/b5ype/). Significance of group-level parameters was estimated by calculating whether the 95% highest density intervals (HDIs) on the posterior distributions of the correlation coefficients ρ_*MiMj*_ overlapped with zero, which is a Bayesian analogue of a frequentist confidence interval because it is the smallest interval containing 95% of the Markov chain Monte Carlo samples (Kruschke, 2014).

We complemented the HMeta-d analyses for metacognitive efficiency with nonhierarchical Pearson’s r correlations and paired *t* tests for magnitude of judgments and task performance. For paired *t* tests, outliers were detected using three tests: leverage, RSS, and Cook’s distance. When necessary, Bonferroni corrections were applied.

## Results

### Type I Performance

**Figure 2 fig2:**
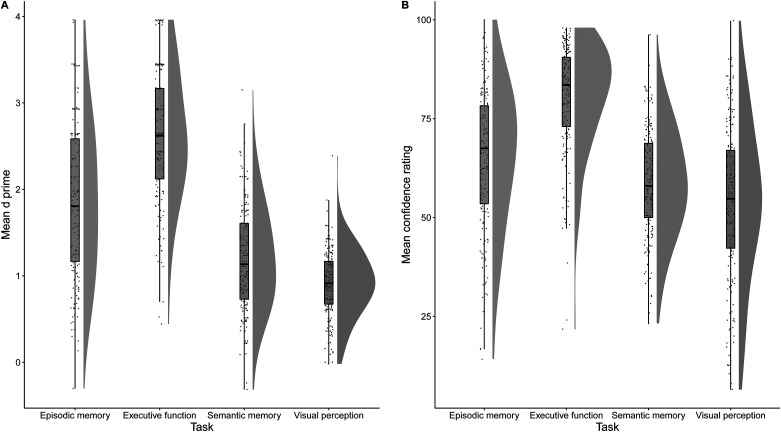
A, Raincloud plots (Allen, Poggiali, Whitaker, Marshall, & Kievit, 2019) for *d*′ for the four tasks. B, Raincloud plots for confidence level for the four tasks.

We assessed task performance using type I *d*′. This index was calculated for each participant and each task (see Figure 2A for mean and confidence intervals). For these analyses a Bonferroni correction was used providing a significance threshold of α = .05/6 = 0.008. Paired *t* tests showed that performance on the executive function task (*M* = 2.58; *SD* = 0.74) was better than the episodic memory task (*M* = 1.84; *SD* = 0.88), *t*(180) = 9.42, *p* < .001, *d*_*z*_ = 0.70, semantic memory task (*M* = 1.19; *SD* = 0.60), *t*(180) = 22.71, *p* < .001, *d*_*z*_ = 1.69, and visual perception task (*M* = 0.92; *SD* = 0.39), *t*(180) = 30.26, *p* < .001, *d*_*z*_ = 2.25. The episodic memory task was also better performed than the semantic memory task, *t*(180) = 9.32, *p* < .001, *d*_*z*_ = 0.69, and the visual perception task, *t*(180) = 13.09, *p* < .001, *d*_*z*_ = 0.97. Finally, the semantic memory task was better performed than the visual perception task, *t*(180) = 4.98, *p* < .001, *d*_*z*_ = 0.37.[Fig-anchor fig2]

We next examined intersubject correlations in first-order performance across tasks. Table 1 summarizes Pearson correlation coefficients between *d*′ values. These analyses revealed a positive correlation between episodic and semantic memory performance, *r* = .23, *p* = .002. Executive function performance was also positively correlated with semantic memory performance, *r* = .27, *p* < .001, and visual perception performance, *r* = .21, *p* < .001. However, correlations between other task performance pairings (visual perception and episodic memory; executive function and episodic memory; semantic memory and visual perception) were not significant after correcting for multiple comparisons.[Table-anchor tbl1]

### Confidence Level

Mean confidence judgments were calculated for each participant and each task (Figure 2B). The pattern of results for confidence judgments was similar to that for task performance. Paired *t* tests (corrected for multiple comparisons) showed people were more confident overall on the executive function task than the episodic memory task, *t*(180) = 10.04, *p* < .001, *d*_*z*_ = 0.75, the semantic memory task, *t*(180) = 18.73, *p* < .001, *d*_*z*_ = 1.39, and the visual perception task, *t*(180) = 18.10, *p* < .001, *d* = 1.35. The episodic memory task was also judged with higher confidence than the semantic memory task, *t*(180) = 4.71, *p* < .001, *d*_*z*_ = 0.35, and the visual perception task, *t*(180) = 6.30, *p* < .001, *d*_*z*_ = 0.47. Finally, the semantic memory task was judged with higher confidence than the visual perception task, *t*(180) = 3.37, *p* < .001, *d*_*z*_ = 0.25.

To estimate domain-general influences on confidence level, we computed correlations between average confidence levels across tasks (see Table 2). We observed a significant correlation between confidence levels across all tasks after correction for multiple comparisons (all *p* < .008, with *r* ranging from 0.21 to 0.39; the exception was a trend-level correlation between visual perception and episodic memory), suggesting that the more participants report high confidence in one task, the more they report high confidence in another task.[Table-anchor tbl2]

### Metacognitive Efficiency

**Figure 3 fig3:**
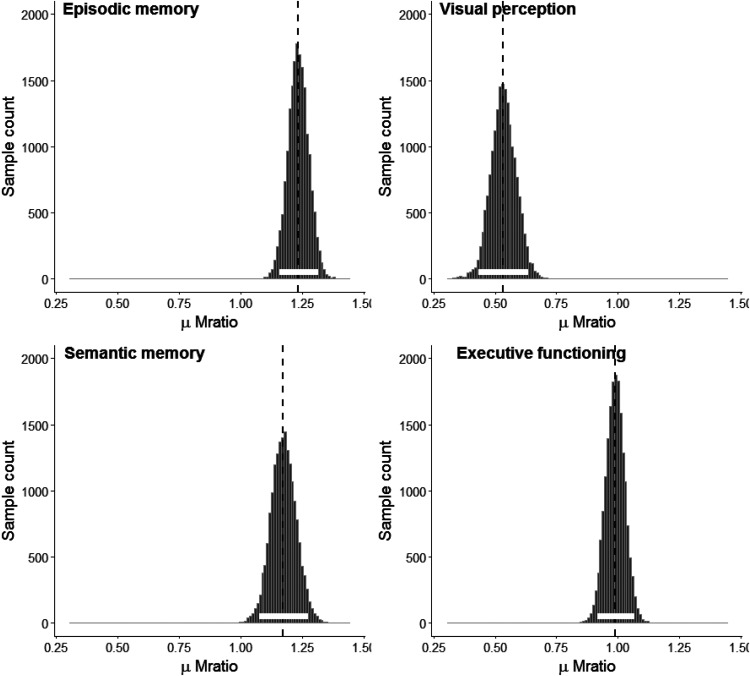
Posterior distributions over μ Mratio (meta-*d*′/*d*′ ratio) for the episodic memory, visual perception, semantic memory, and executive functioning tasks.

To estimate metacognitive efficiency, we estimated the group meta-*d*′/*d*′ ratio for each task (see Figure 3). According to the overlap of 95% HDIs, metacognitive efficiencies were similar for the two memory tasks, which in turn were greater than both the executive function and visual perception tasks (for means and HDIs related to the difference distributions for each comparison see Table 3). Executive function metacognitive efficiency was also greater than visual perceptual metacognitive efficiency.[Fig-anchor fig3][Table-anchor tbl3]

**Figure 4 fig4:**
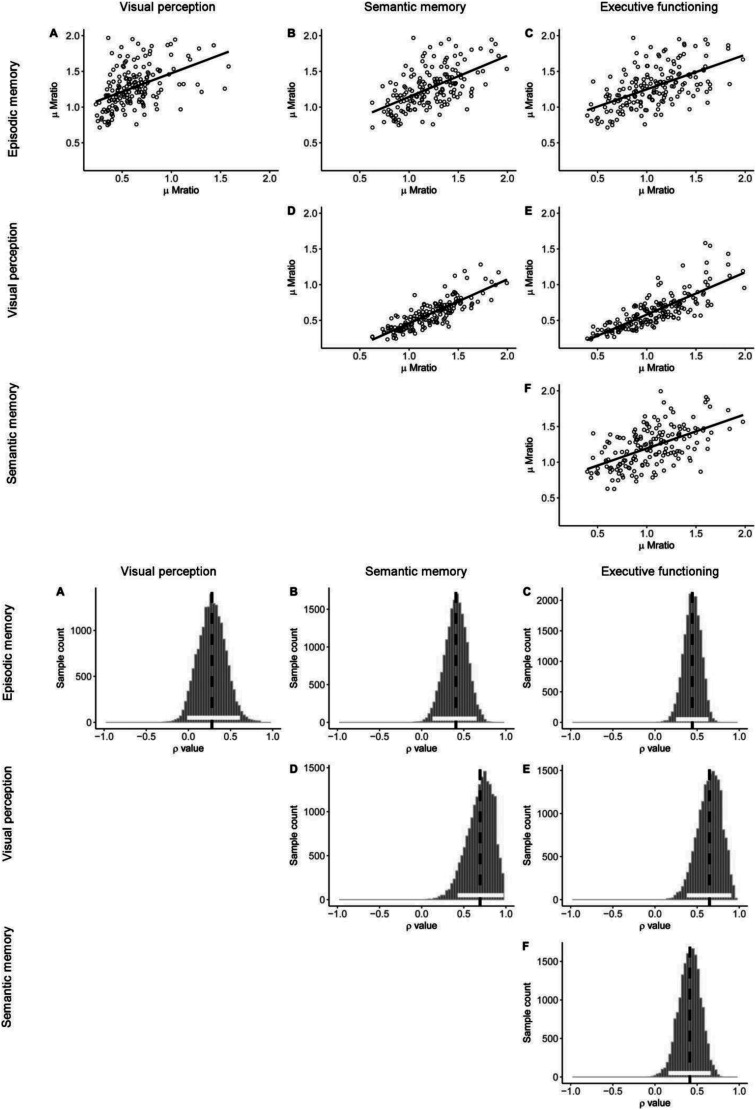
A, Single-subject parameter estimates from the hierarchical model of meta-*d*′/*d*′ and Pearson correlations between meta-*d*′/*d*′ estimates across the four tasks. B, Posterior distributions over ρ for each entry in the covariance matrix determining the correlations between meta-*d*′/*d*′ across the four tasks.

To evaluate domain-general contributions to metacognitive efficiency, we estimated correlations between all four task pairings within the hierarchical model. These correlations are estimated at the group level from the variance-covariance matrix. Figure 4B presents posterior distributions over each cross-task correlation parameter and associated 95% HDIs are presented in Table 4. Figure 4A visualizes the relationships between single-subject meta-*d*′/*d*′ values estimated within the hierarchical model. Critically, 95% HDIs on the posterior correlation coefficients for five of six task pairings did not overlap zero, suggesting substantial covariance in metacognitive efficiency across domains. This was also the case for task pairings for which we did not observe correlations in task performance (e.g., visual perception and semantic memory; Table 1), suggesting it is unlikely to be an artifact of covariance in first-order capacity. Only the HDI for the correlation between visual perception task and episodic memory task (ρ = 0.28; HDI = [−0.03, 0.60]) overlapped zero, indicating a lack of cross-task correlation.[Fig-anchor fig4][Table-anchor tbl4]

Although the current study has few trials per task, for completeness we nonetheless performed nonhierarchical estimation of subject-specific meta-*d*′ to calculate a meta-*d*′/*d*′ ratio per participant and per task. We excluded nine participants with very low performance (*d*′ < 0.10) in one of the four tasks. Then we performed Pearson’s correlations for metacognitive efficiency across tasks (see Table 5). When controlling for multiple comparisons, we found positive correlations for meta-*d*′/*d*′ across visual perception and semantic memory and across visual perception and executive function.[Table-anchor tbl5]

## Discussion

The present study compared RCJs across four cognitive tasks to quantify a potential domain-general metacognitive resource. We focused on both confidence level and metacognitive efficiency. Our study goes beyond previous studies by using a large sample to increase reliability, using four distinct 2AFC tasks to avoid problems that arise when comparing different task formats and using a hierarchical estimation of meta-*d*′/*d*′ (and covariance parameters) that facilitated efficient estimation of group-level correlation parameters.

We reproduced previous findings on the domain generality of metacognitive bias using a confidence level (e.g., Ais et al., 2016). Except for a trend between episodic memory and visual perception, we found that the tendency to report high confidence in one task is correlated with the tendency to report high confidence in another task, suggesting domain-general contributions to overall confidence level. These results are in line with judgments of confidence being biased by domain-general contextual factors such as mood (see Ais et al., 2016 for influences of optimism on bias) and psychiatric symptomology (see Rouault, Seow, Gillan, & Fleming, 2018 in perceptual decision making).

Our study also allowed us to estimate the extent of across-task stability in metacognitive efficiency by estimating the parameters of a covariance matrix governing the association between meta-*d*′/*d*′ values in a hierarchical framework. We found substantial shared variance in meta-*d*′/*d*′ across tasks, with five of six correlation parameters deviating from zero. Because the meta-*d*′/*d*′ measure controls for influences of task performance, this result suggests a substantial shared variance in metacognitive efficiency and is consistent with a domain-general resource supporting metacognition. Critically, these correlations were obtained even for pairs of tasks that did not show correlations in first-order performance (i.e., for semantic memory and visual perception; for episodic memory and executive function). This suggests that correlations in metacognitive efficiency are unlikely to be driven by covariance in task performance.

The one 95% HDI that did overlap zero, for the correlation between episodic memory and visual perception, still showed a substantial probability mass above zero, suggesting uncertainty around the proportion of shared variance, rather than an absence of correlation (HDI = [−0.03, 0.60]). Although our findings are less clear regarding these two tasks, a recent study (Lee et al., 2018) suggested a positive relationship between metacognitive sensitivity for short-term memory and visual perception when comparing 2AFC tasks using a large sample size (100 participants) and a larger number of trials (120 trials). The correlation they found was very close to the one we estimated here (*r* = .31 and *r* = .28).

Our results on shared variance in metacognitive efficiency across tasks thus suggest the involvement of a common resource in metacognitive sensitivity across domains. Nevertheless, it seems that the involvement of this common resource differed across tasks, with variation in the strength of cross-task correlations (from 0.28 to 0.69). From this perspective, general metacognition explains between 7% and 48% of the variance in cross-task meta-*d*′/*d*′ estimates (i.e., *r*^2^ coefficient). Because this range is large, it supports the idea that both domain-general and domain-specific processes are at play in metacognition. Recent work has indeed found common and distinct brain areas tracking confidence across recognition memory and visual perceptual metacognition tasks (Morales et al., 2018), supporting the idea that both domain-specific and domain-general processes may influence the sensitivity of metacognitive judgments. Moreover, the contribution of a putative global resource may differ according to the cognitive domain.

Comparing the highest cross-task correlation (semantic memory and visual perception) with the lowest (episodic memory and visual perception) is especially interesting. We will briefly highlight a potential reason for this difference in the use of domain-general metacognition. In the visual perception task, there is an objective level of task difficulty—the difference in terms of number of dots between the two stimuli. In the semantic memory task, difficulty varies in a more subjective way: for instance, people are more likely to know a very famous actor compared with a less well-known one. However, because people share representations about general knowledge (e.g., Juslin, 1993), this variation could also create shared knowledge about task difficulty and therefore a quasiobjective level (or at least an intersubjective or a consensual level; Koriat, 2008). As such, a putative domain-general metacognitive resource could reflect the ability to build metalevel representations of task difficulty to infer confidence. We would therefore expect that the more task difficulty can be easily inferred across two tasks (e.g., from shared experimental cues, see Barthelmé & Mamassian, 2010), the more metacognitive efficiency would also correlate. Conversely, if one task has an easily available difficulty signal and another task did not, we would expect a lower cross-task correlation for metacognitive efficiency: the ability to infer task difficulty is less useful for the second task. We suggest that such a lack of correlation occurs in the episodic memory task because there is less intersubject consensus regarding task difficulty, and such domain-general cues are less readily available.

Our findings are also consistent with a second-order model that proposes that a common algorithm for second-order inference may be engaged across domains (Fleming et al., 2017). As such, shared aspects of the state space, such as motor responses being shared across tasks (Faivre et al., 2018), can increase the prevalence of domain-general metacognition. Another driver to global metacognition would be the ability to generalize priors from one task to another, such as between two memory tasks, or two perceptual tasks (Rouault et al., 2018). Although some cross-domain cues and processes influencing bias have been identified (as described above), further research should focus on identifying domain-general processes influencing metacognitive efficiency.

When analyzing our data using a nonhierarchical estimation of cross-task correlations, only two of five correlations remained significant. This is likely due to the low number of trials in this experiment, and we suggest that the hierarchical model is more powerful and accurate in this context (see *Method* section). To confirm this intuition, we carried out simulations to compare the power of hierarchical and nonhierarchical estimation procedures in recovering cross-task correlations in metacognitive efficiency (see online supplemental material). Simulated data were generated using the variance-covariance matrix and parameters estimated from data from the current experiment. When analyzing these data using both hierarchical and nonhierarchical estimations of cross-task correlations, we found that the hierarchical model estimations achieved a closer match to the ground truth correlations than the nonhierarchical fits for a low number of trials (*N* = 40), a difference that was not seen when conducting parameter recovery simulations with a higher number of trials (*N* = 400). In the present work, we opted to use a large number of participants and several cognitive tasks to study a breadth of cross-task correlations and isolate a domain-general resource. However, this approach was at the expense of having fewer trials per task. It will be important to replicate our findings with a higher number of trials to strengthen conclusions regarding the involvement of a domain-general resource for metacognitive efficiency.

Finally, as in previous studies (e.g., Morales et al., 2018), we found that metacognitive efficiency was better for memory (for both episodic and semantic memory tasks in the present study) compared with visual perception. Here we consider potential explanations of this difference. One potential possibility is that the one-dimensional SDT model that underpins the modeling of metacognitive efficiency is less appropriate for memory compared with perception tasks because memory decisions are presumably made by matching a target to a sample in a high-dimensional space. How confidence is formed in such a situation, and how the link between confidence and accuracy should be modeled relative to SDT-observer predictions therefore remains an open question (van den Berg, Yoo, & Ma, 2017). This may especially be the case for episodic memory decisions, which have been proposed to be influenced both by a familiarity process accommodated by classical SDT and an all-or-none recollection process (e.g., Yonelinas, 1994, 2002). Another possibility is that control processes exert a greater influence on confidence in the memory compared with perceptual task. In memory, metacognitive beliefs are important in regulating attempts to retrieve information: Participants are more likely to engage in a search if they believe they can recall the information (Nelson & Narens, 1994). Thus, a positive feedback loop might ensue in which good metacognitive sensitivity is used to guide memory search, which in turn may further increase measured metacognitive sensitivity: If one knows that she can remember the answer, she will engage a search in memory, which is more likely to lead to successful remembering (compared with a situation with no active search in memory). Conversely, a belief that one cannot remember a target would lead to weaker memory search and the increased likelihood of an incorrect response. However, such a belief would be metacognitively informative for these incorrect responses (i.e., “this response should be incorrect because I did not search in memory”), therefore increasing metacognitive sensitivity. We suggest that such processes are less likely to occur in the case of visual perception, which would point to a unique variance component associated with metamemory. However, as also suggested by previous work (Morales et al., 2018), both domain-specific processes and a more domain-general resource may make independent contributions to confidence formation.

To conclude, we find that contrary to previous results, both metacognitive bias (measured by confidence level) and metacognitive efficiency share common resources across domains. This observation of a domain-general signature of metacognitive efficiency was obtained after ensuring that task structures were similar across domains (2AFC), that experimental power was sufficient, and that performance-controlled measures of metacognition were used (meta-*d*′/*d*′). The percentage of explained variance, however, suggests that both domain-specific and domain-general resources are involved in metacognitive efficiency, which is consistent with previous neuroimaging data (Morales et al., 2018) and models of confidence formation (Fleming et al., 2017). It also suggests that the use of a global resource may differ according to the evaluated domain. Nevertheless, this lends support to the idea that training metacognitive efficiency in one domain can enhance metacognitive efficiency in another domain (Carpenter et al., 2018). Such transfer effects on metacognition may have important implications for education and rehabilitation programs because they offer a pathway toward generalized improvements of awareness of abilities (or disabilities). Although domain-general cues have been identified for biases in confidence judgment (i.e., anchoring effects, confirmation bias), the source(s) of domain-generality in metacognitive efficiency has received less attention. Further work should focus on identifying the types of processes which influence metacognitive efficiency across domains. A second question of interest is understanding sources of variation in cross-task correlation, which may indicate that some tasks are less likely than others to rely on a domain-general metacognitive resource.

## Context

This experiment is the first in a series conducted as part of Audrey Mazancieux’s doctoral research program, the details of which can be found on OSF. The aim of the research program is to assess whether there are domain-general resources contributing to metacognition. More broadly, the research straddles a well-established metacognitive tradition grounded in memory research (hence the focus on memory) and the more recent field inspired by psychophysical experiments examining metaperception (hence the signal-detection inspired modeling approach). The research reported here benefited from a collaborative visit by A.M. to S.M.F.’s laboratory in 2018 after the study had been preregistered and the data had been collected. This greatly influenced the choice of hierarchical modeling approach, leading to the changes between our preregistered analysis plans and the results presented here.

## Supplementary Material

10.1037/xge0000746.supp

## Figures and Tables

**Table 1 tbl1:** Pearson Correlation Coefficients, Confidence Intervals, and p Values for Correlations in Task Performance Between Pairs of Tasks

Variables	Performance (d’)			
	Episodic memory	Visual perception	Semantic memory	Executive function
Episodic memory		*r* = .04 [−0.11, 0.18]	***r* = .23 [0.09, 0.37]**	*r* = .16 [0.02, 0.30]
		*p* = .638	***p* = .002**	*p* = .030
Visual perception			*r* = −0.08 [−0.23, 0.06]	***r* = .25 [0.11, 0.39]**
			*p* = .258	***p* < .001**
Semantic memory				***r* = .25 [0.11, 0.38]**
				***p* < .001**
Executive function				
*Note*. Alpha threshold is .008. Significant tests are in bold.				

**Table 2 tbl2:** Pearson Correlation Coefficients, Confidence Intervals, and p Values for Paired Correlations of Confidence Levels Across Tasks

Variables	Correlations in confidence level			
	Episodic memory	Visual perception	Semantic memory	Executive functioning
Episodic memory		*r* = .19 [0.05, 0.33]	***r* = .34 [0.21, 0.46]**	***r* = .21 [0.06, 0.34]**
		*p* = .009	***p* < .001**	***p* = .005**
Visual perception			***r* = .39 [0.27, 0.52]**	***r* = .36 [0.23, 0.48]**
			***p* < .001**	***p* < .001**
Semantic memory				***r* = .37 [0.23, 0.49]**
				***p* < .001**
Executive functioning				
*Note*. Alpha threshold is .008. Significant tests are in bold.				

**Table 3 tbl3:** Means and HDIs of the Posteriors of the Difference Between μ Mratio Distributions for Each Task Pairing

Variables	Difference distributions between group-level meta-*d*′/*d*′ estimates			
	Episodic memory	Visual perception	Semantic memory	Executive function
Episodic memory		**0.84** [0.72, 0.97]	0.05 [−0.01, 0.11]	**0.22** [0.17, 0.28]
Visual perception			**0.79** [0.68, 0.91]	**0.62** [0.51, 0.75]
Semantic memory				**0.17** [0.11, 0.23]
Executive function				
*Note*. Only the difference distribution between episodic memory and semantic memory overlaps with 0, indicating no significant difference between tasks. Significant tests are in bold.				

**Table 4 tbl4:** Means and HDIs of the Distribution of Posteriors of the ρ Value for Each Task Pairing. Only the HDI for the Correlation Between Episodic Memory and Visual Perception Overlap With 0, Indicating a Lack of Cross-Task Correlation

Variables	Group-level correlations in meta-*d*′/*d*′			
	Episodic memory	Visual perception	Semantic memory	Executive function
Episodic memory		0.28 [−0.03, 0.60]	**0.41** [0.14, 0.66]	**0.44** [0.24, 0.63]
Visual perception			**0.69** [0.36, 0.94]	**0.65** [0.35, 0.89]
Semantic memory				**0.41** [0.16, 0.65]
Executive function				
*Note*. Significant tests are in bold.				

**Table 5 tbl5:** Pearson Correlation Coefficients, Confidence Intervals, and p Values for Correlations in Individual meta-d′/d′ Between Pairs of Tasks

Variables	Correlations in individual meta-*d*′/*d*′ estimates			
	Episodic memory	Visual perception	Semantic memory	Executive function
Episodic memory		*r* = .10 [−0.05, 0.24]	*r* = .05 [−0.10, 0.19]	*r* = .18 [0.04, 0.33]
		*p* = .212	*p* = .516	*p* = .015
Visual perception			***r* = .21 [0.06, 0.35]**	***r* = .25 [0.11, 0.39]**
			***p* = .006**	***p* < .001**
Semantic memory				*r* = .12 [−0.03, 0.26]
				*p* = .106
Executive function				
*Note*. Alpha threshold is .008. *N* = 172. Significant tests are in bold.
