# Supplementary material for: Is There a G Factor for Metacognition? Correlations in Retrospective Metacognitive Sensitivity Across Tasks
Source: J Exp Psychol Gen. 2020 Mar 19;149(9):1788–99. doi: 10.1037/xge0000746 (PMC7397761; doi:10.1037/xge0000746)
Supplement: Supplementary file 1 [file xge0000746Supplementary.docx]

**Supplementary materials**

**Comparison between hierarchical and non hierarchical models**

We carried out simulations to compare the power of hierarchical and non-hierarchical estimation procedures in recovering cross-task correlations in metacognitive efficiency. Simulated data were generated using the variance-covariance matrix and parameters estimated from data from the current experiment. We used the metad_sim function from the HMeta-d toolbox to generate confidence rating data (this function simulates confidence rating data from the meta-d’ model with pre-specified levels of meta-d’/d’; see Fleming, 2017, for further details).

Two types of dataset were generated: one with 40 trials per task/subject (as in the current study) and one with 400 trials per task. As in the current experiment, we simulated data for 181 participants and for 11 distinct confidence rating levels. For each participant and each task, meta-d’/d’ ratios were sampled from a multivariate Gaussian distribution (using the means and covariance matrix obatined from the current study) and d’ values were sampled from a normal Gaussian distribution (again using the parameters from the current study). Then, simulated confidence ratings were generated from each participant’s d’ and meta-d’. Decision and confidence criteria were fixed across tasks and participants. Code and generated data are available on OSF (<https://osf.io/b5ype/>).

*Simulation parameters*

- Group-d’: EM = 1.84; VP = 1.19; SM = 0.92; EF = 2.58

- Type 1 σ: EM = 0.88; VP = 0.60; SM = 0.36; EF = 0.74

- Group-Mratio: EM = 1.23; VP = 0.53; SM = 1.17; EF = 0.99

- Type 2 sigma: EM =0.31; VP = 0.54; SM = 0.36; EF = 0.39

- ρ: EM/VP = 0.28; EM/SM = 0.41; EM/EF = 0.44; SM/VP = 0.69; SM/EF = 0.68; VP/EF = 0.41

- c: 0

- c_2, “S1”_: (-2, -1.8, -1.6, -1.4, -1.2, -1, -0.8, -0.6, -0.4, -0.2)

- c_2, “S2”_: (0.2, 0.4, 0.6, 0.8, 1, 1.2, 1.4, 1.6, 1.8, 2)

*Simulating data with 40 trials per task*

First, we estimated group-level parameters from the simulated data (mean and covariance of meta-d’/d’ ratios across tasks) using the hierarchical Bayesian model (Fleming, 2017). The mean and HDIs of group-level posterior distributions over the cross-task correlation parameters are presented in the Figure. The red vertical line showed the ground-truth correlations, and the black line shows the recovered parameter. 5 out of 6 of the recovered correlations were significantly above zero, as was the case for the actual data.


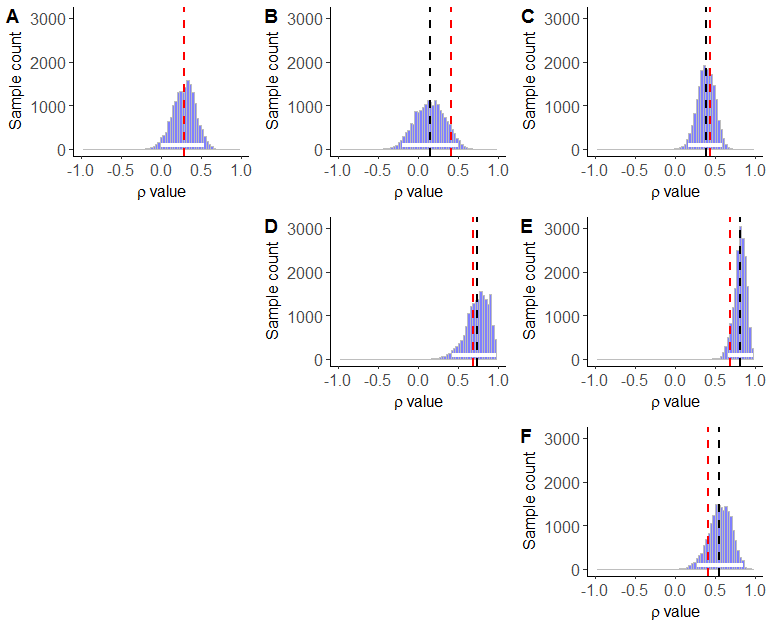


**Figure S2.** Posterior distributions over ρ for each entry in the covariance matrix determining the correlations between meta-*d’/d’* across the four tasks. N = 40 trials. Red lines are ground truth correlations and black lines are mean correlations estimated by the model.

Second, we analysed the same simulated data by estimating one meta-d’ / d’ ratio per participant and per task using a single-subject Bayesian model (Fleming, 2017). We excluded 13 simulated participants with very low performance (d’ < 0.10) in one of the four tasks. Then, we performed Pearson’s correlations for estimated metacognitive efficiency across tasks.

**Table S2**. Pearson correlation coefficients, confidence intervals, and p values for paired correlations of simulated meta-d’/d’ ratios across tasks. Simulated participants with d’ lower than 0.1 were excluded. N = 168. Alpha threshold is .008.

|  | Individual Mratio | | | |
| --- | --- | --- | --- | --- |
|  | Episodic memory | Visual perception | Semantic memory | Executive functioning |
| Episodic memory |  | *r* = 0.11 [-0.04, 0.26]  *p* = .147 | *r* = 0.02 [-0.14, 0.17]  *p* = .838 | *r* = 0.13 [-0.02, 0.28] *p* = .083 |
| Visual perception |  |  | *r* = 0.07 [-0.08, 0.22] *p* = .367 | *r* = 0.17 [0.02, 0.31] *p* = .028 |
| Semantic memory |  |  |  | ***r* = 0.40 [0.26, 0.52] *p* < .001** |
| Executive functioning |  |  |  |  |

When comparing correlations estimated using single-subject estimations of meta-d’/d’ and those obtained within the hierarchical model, the latter are closer to the ground truth correlations (see Table S3) except for the correlation between executive function and semantic memory. 5 out of 6 correlations estimated within the hierarchical model were significantly above zero, compared to only 1 out of 6 correlations using the single-subject approach. Finally, correlations estimated from single-subject values tended to be lower than those estimated in the hierarchical model (Table S2).

**Table S3**. Comparisons of values for “true” correlations, correlations estimated within the hierarchical model and correlations calculated from single-subject estimations. N = 181 subjects and N = 40 trials.

|  |  |  |  |
| --- | --- | --- | --- |
|  | Comparisons | | |
|  | "True" correlation | Hierarchical estimation | Single-subject estimation |
| EM / VP | 0.28 | 0.28 | 0.11 |
| EM / SM | 0.41 | 0.15 | 0.02 |
| EM / EF | 0.44 | 0.38 | 0.13 |
| VP / SM | 0.69 | 0.73 | 0.07 |
| VP / EF | 0.68 | 0.81 | 0.17 |
| SM / EF | 0.41 | 0.55 | 0.40 |
|  |  |  |  |

*Simulating data with 400 trials per task*

We next repeated the same simulation and parameter recovery process when generating 400 simulated trials per task/subject. Results from the hierarchical estimation are presented in Figure S3, using the same conventions as before.


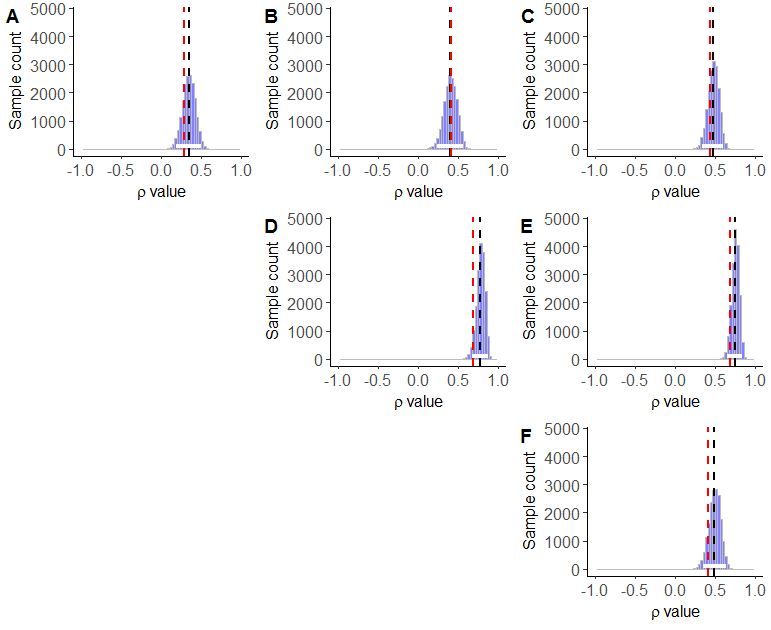


**Figure S3.** Posterior distributions over ρ for each entry in the covariance matrix determining the correlations between meta-*d’/d’* across the four tasks. N = 400 trials. Red lines are ground truth correlations and black lines are mean correlations estimated by the model.

We next estimated one meta-d’ / d’ ratio per participant and per task using a single-subject Bayesian model (Fleming, 2017). As in the 40-trial simulation, we excluded 13 simulated participants with very low performance (d’ < 0.10) in one of the four tasks were excluded. Cross-task correlations are presented in Table S4.

**Table S4**. Pearson correlation coefficients, confidence intervals, and p values for paired correlations of simulated meta-d’/d’ ratios across tasks. Simulated participants with d’ lower than 0.1 were excluded. N = 168 subjects and N = 400 trials. Alpha threshold is .008.

|  | Individual Mratio | | | |
| --- | --- | --- | --- | --- |
|  | Episodic memory | Visual perception | Semantic memory | Executive functioning |
| Episodic memory |  | ***r* = 0.24 [0.10, 0.38]**  ***p* = .002** | ***r* = 0.30 [0.16, 0.43]**  ***p* < .001** | ***r* = 0.42 [0.29, 0.54] *p* < .001** |
| Visual perception |  |  | ***r* = 0.59 [0.44, 0.66]**  ***p* < .001** | ***r* = 0.60 [0.50, 0.69] *p* < .001** |
| Semantic memory |  |  |  | ***r* = 0.41 [0.28, 0.53] *p* < .001** |
| Executive functioning |  |  |  |  |

**Table S5**. Comparisons of values for “true” correlations, correlations estimated within the hierarchical model and correlations calculated from single-subject estimates. N = 181 subjects and N = 400 trials.

|  |  |  |  |
| --- | --- | --- | --- |
|  | Comparisons | | |
|  | "True" correlation | Hierarchical estimation | Single-subject estimation |
| EM / VP | 0.28 | 0.34 | 0.24 |
| EM / SM | 0.41 | 0.40 | 0.30 |
| EM / EF | 0.44 | 0.46 | 0.42 |
| VP / SM | 0.69 | 0.77 | 0.59 |
| VP / EF | 0.68 | 0.74 | 0.60 |
| SM / EF | 0.41 | 0.49 | 0.41 |
|  |  |  |  |

Now, in contrast to 40 trials, the comparison between correlations recovered using single-subject estimates of meta-d’/d’ and those recovered within the hierarchical model showed that all values were closer to the “true” correlations (see Table S5). Therefore, we conclude that the benefit of the hierarchical approach is likely to be particularly pronounced when low trial numbers lead to individual meta-d’/d’ estimates to be unreliable (see also Fleming, 2017).

**Preregistered analyses**

*Estimating metacognitive bias using difference scores*

The difference between mean confidence and mean performance was calculated for each participant and each task. As the performance scale (from 50% to 100% of performance) was different from the confidence scale (0% to 100%) we transformed the confidence scale. Therefore, 0% was 50%, 10% was 55%, 20% was 60% and so on. Here, a negative score refers to underestimation of performance whereas a positive score refers to an overestimation. Bonferroni corrected single sample t-tests were used (α = 0.05/6 = 0.008); revealing that confidence tended to be higher than task performance (episodic memory: *M* = 0.015, *SD* = 0.084; semantic memory: *M* = 0.071, *SD* = 0.089; visual perception: *M* = 0.094, *SD* = 0.116; all *p* < .05) except for the executive functioning task which was neither underestimated nor overestimated (*M* = 0.001, *SD* = 0.057, *t*(180) = 0.28, *p* = .778, *d_z_* = 0.02).

We also compared these difference scores across task using Bonferroni corrected paired t-tests (α = 0.05/6 = 0.008). Analyses showed that metacognitive bias was the same in the executive functioning task and the episodic memory task, *t*(180) = 2.16, *p* = .032, *d_z_* = 0.17, whereas it was lower in the executive functioning task compared to the semantic memory task, *t*(180) = 10.28, *p* < .001, *d_z_* = 0.80, and than visual perception task, *t*(180) = 11.77, *p* < .001, *d_z_* = 0.92. The episodic memory task has also a lower bias than the semantic memory, *t*(180) = 7.83, *p* < .001, *d_z_* = 0.61, and than the visual perception task, *t*(180) = 9.50, *p* < .001, *d_z_* = 0.74. Finally, the bias for the visual perception task was higher than that for the semantic memory task, *t*(180) = 2.67, *p* = .008, *d_z_* = 0.21. Overall, overconfidence in performance tended to be higher in tasks with lower performance.

In order to estimate domain-general and domain-specific influences on these difference scores, we computed correlations across tasks (see Table below). All scores significantly correlated which each other (all *p* < .001 and *r* ranging from 0.27 to 0.42) suggesting that the more a participant overestimates their performance in one task, the more they overestimate their performance in another task.

**Table S6**. Pearson correlation coefficients, confidence intervals, and p values for paired correlations of metacognitive bias (mean confidence - mean performance) across tasks. Alpha threshold is .008.

|  | Metacognitive bias | | | |
| --- | --- | --- | --- | --- |
|  | Episodic memory | Visual perception | Semantic memory | Executive functioning |
| Episodic memory |  | ***r* = 0.41 [0.28, 0.52] *p* < .001** | ***r* = 0.37 [0.24, 0.49] *p* < .001** | ***r* = 0.35 [0.21, 0.47] *p* < .001** |
| Visual perception |  |  | ***r* = 0.42 [0.29, 0.53] *p* < .001** | ***r* = 0.42 [0.29, 0.53] *p* < .001** |
| Semantic memory |  |  |  | ***r* = 0.27 [0.13, 0.40] *p* < .001** |
| Executive functioning |  |  |  |  |

*Metacognitive sensitivity using AUROC2*

We computed the area under the type 2 ROC curve (AUROC2) for each participant and each task. The Type 2 ROC curve represents the relationship between performance (i.e., type 1 decision as correct or incorrect) and confidence. AUROC2 ranges between 0.5 and 1 where 0.5 refers to chance-level discrimination between correct and incorrect answers and 1 refers to perfect discrimination.

We excluded 17 participants from these analyses because they gave 100% correct answers in one of the 4 tasks. As we were interested in the comparison of AUROC2 values across all task pairs the significance threshold for these analyses was α = 0.05/6 = 0.008. Paired t-tests were conducted across tasks and showed that executive functioning AUROC2 was higher than episodic memory AUROC2, *t*(163) = 2.69, *p* = .008, *d_z_* = 0.21, semantic memory AUROC2, *t*(163) = 8.42, *p* < .001, *d_z_* = 0.66, and visual perception AUROC2, *t*(163) = 19.92, *p* < .001, *d_z_* = 1.56. Furthermore, episodic memory AUROC2 was higher than semantic memory AUROC2, *t*(163) = 6.07, *p* < .001, *d_z_* = 0.47 and visual perception AUROC2, *t*(163) = 16.95, *p* < .001, *d_z_* = 1.32. Finally, semantic memory AUROC2 was higher than visual perception AUROC2, *t*(163) = 12.78, *p* < .001, *d_z_* = 1.00. As further discussed in the main text, these differences in metacognitive sensitivity are potentially confounded by differences in first-order performance (d’), hence why we decided to use meta-d’/d’ as our primary measure of metacognitive efficiency when analyzing cross-task correlations.


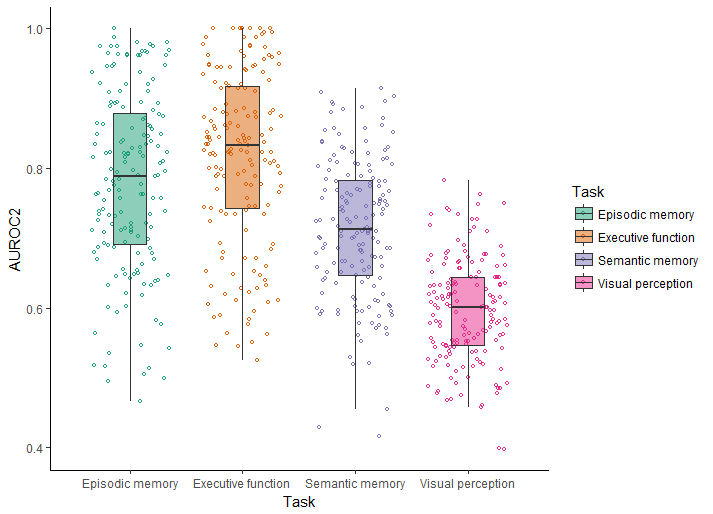


**Figure S4**. AUROC2 values per participant and per tasks.

For completeness, we also examined correlations in AUROC2 across tasks (see Table S7). We found a significant correlation between AUROC2 values estimated from the episodic and semantic memory tasks, *r* = 0.23, *p* = .003, but other correlations did not reach significance.

**Table S7**. Pearson correlation coefficients, confidence intervals, and p values for paired correlations of AUROC2 across tasks. Alpha threshold is .008.

|  | Metacognitive sensitivity (AROC) | | | |
| --- | --- | --- | --- | --- |
|  | Episodic memory | Visual perception | Semantic memory | Executive functioning |
| Episodic memory |  | *r* = 0.14 [-0.01, 0.29]  *p* = .066 | ***r* = 0.23 [0.08, 0.37] *p* = .003** | *r* = 0.13 [-0.03, 0.27] *p* = .106 |
| Visual perception |  |  | *r* = 0.13 [-0.03, 0.28] *p* = .010 | *r* = 0.07 [-0.09, 0.22] *p* = .385 |
| Semantic memory |  |  |  | *r* = 0.04 [-0.11, 0.19] *p* = .609 |
| Executive functioning |  |  |  |  |
